# Health App Use Among Individuals With Symptoms of Depression and Anxiety: A Survey Study With Thematic Coding

**DOI:** 10.2196/mental.7603

**Published:** 2017-06-23

**Authors:** Caryn Kseniya Rubanovich, David C Mohr, Stephen M Schueller

**Affiliations:** ^1^ Center for Behavioral Intervention Technologies Department of Preventive Medicine Northwestern University Chicago, IL United States

**Keywords:** mHealth, eHealth, mobile health, depression, anxiety

## Abstract

**Background:**

Researchers have largely turned to commercial app stores, randomized trials, and systematic reviews to make sense of the mHealth landscape. Few studies have approached understanding by collecting information from target end users. The end user perspective is critical as end user interest in and use of mHealth technologies will ultimately drive the efficacy of these tools.

**Objective:**

The purpose of this study was to obtain information from end users of mHealth technologies to better understand the physical and mental health apps people use and for what purposes.

**Methods:**

People with depressive or anxious symptoms (N=176) seeking entry into a trial of mental health and well-being apps for Android devices completed online questionnaires assessing depression and anxiety (Patient Health Questionnaire-9 and Generalized Anxiety Disorder-7), past and current mental health treatment-seeking behavior, overall mobile device use, and use of mobile health apps. Participants reported the physical health and mental health apps on their devices and their reasons for using them. Data were extracted from the participant self-reports and apps and app purposes were coded in order to categorize them.

**Results:**

Participants were largely white, middle-aged females from the Midwest region of the United States recruited via a health care organization and Web-based advertising (135 female, 41 male, mean age 38.64 years, age range 19-75 years.) Over three-quarters (137/176, 77.8%) of participants indicated having a health app on their device. The top 3 kinds of apps were exercise, fitness, and pedometers or heart rate monitoring apps (93/176, 52.8%); diet, food, or calorie counting apps (65/177, 36.9%); and mental health/wellness apps (46/177, 26.1%). The mean number of mobile physical and mental health apps on a participant’s phone was 2.15 (SD 3.195). Of 176 participants, 107 (60.8%) specifically reported the top 5 health apps that they used and their purposes. Across the 107 participants, a total of 285 apps were reported, with 139 being unique apps. The majority of these apps were free (129/139, 92.8%). Almost two-thirds of participants (67/107, 62.6%) reported using health apps at least on a daily basis.

**Conclusions:**

Among those seeking support for their well-being via physical and mental health apps, people are using a variety of health apps. These people use health apps on a daily basis, especially free apps. The most common reason for using a health app is to track some health-related data; for mental health apps specifically, training or habit building was the most popular reason. Understanding the end user perspective is important because it allows us to build on the foundation of previously established mHealth research and may help guide future work in mHealth.

**Trial Registration:**

Clinicaltrials.gov NCT02176226; https://clinicaltrials.gov/ct2/show/NCT02176226 (Archived by WebCite at http://www.webcitation.org/6rGc1MGyM)

## Introduction

The landscape of behavioral health care is rapidly being redefined by the creation and deployment of mobile apps for a variety of health conditions. Excitement for the growing field of mHealth comes from multiple stakeholder groups, including patient and provider end users, health care providers, researchers, and funders. This enthusiasm has fueled efforts to design, develop, and deploy mHealth quickly in an attempt to capitalize on users’ interests while adding value to the health care system. Indeed, an overwhelming number of mHealth apps are currently available to the public. One report from the IMS Institute for Healthcare Informatics estimated that a combined 165,000 mHealth apps were available for download between the Apple iTunes and Google Play stores [1]. Most of the downloads, however, are concentrated on a few apps, with only 36 health apps accounting for nearly half of all downloads and the largest group of apps receiving fewer than 5000 downloads. Thus, by exploring the apps that are most commonly downloaded by particular user groups, we may be able to better understand the types of health apps they may be likely to use and examine what those apps do. In this paper, we explore self-reported health app use by people with symptoms of anxiety and depression seeking support from mental health apps.

Researchers have previously sought to make sense of the large number of health apps available and often focused on general or physical health [2-4], although several reviews of mental health apps exist as well [5-12]. These reviews use different approaches, such as (1) exploring commercial app stores [6,10,12], (2) reviewing existing scientific literature [2,5], and (3) a combination of app store and scientific literature reviews [3,4,7-9,11].

Through the exploration of the app marketplaces, researchers can evaluate what people are using by examining download rates, app rankings, or by investigating the apps themselves [3,4,6,7,9,12]. By reviewing scientific literature, researchers can explore the common principles or treatment strategies used in apps and the efficacy of these apps at achieving their clinical targets [5,7,8,11]. By synthesizing controlled trials of health apps, researchers compile evidence exploring their benefits in behavior change, disease management, and symptom reduction [2,5]. Thus, the combination of app store reviews, scientific literature searches, and trials have helped sketch an important understanding of health apps. Nevertheless, there are gaps in what we know of health app use from the end user perspective.

For researchers with a vested interest in evaluating, developing, and deploying mHealth apps, it is critical to understand and include end user experiences and perspectives because they are key stakeholders [13,14]. Unfortunately, studies including the end user perspective are mostly lacking.

One notable exception is a survey conducted by Krebs and Duncan [15] which explored health app use by surveying users of those apps. The study conducted a cross-sectional survey of 1604 people recruited through a quota sampling method. Fitness and nutrition were the most common categories of apps mentioned, with users reporting using health apps for exercise, nutrition, weight management, and checking blood pressure. For example, over half of the people surveyed reported downloading health apps to track physical activity. Additionally, almost one-half reported using health apps to track eating or to lose weight, and one-third reported using health apps to learn exercises. Most users noted learning about health apps via the app store, from a family member or friend, or from Web searches. A large portion of the survey participants (41.27%) said they would not pay for health apps. Overall, this study demonstrates that health apps are widely used; we build upon this work by exploring mental health app use in addition to physical health app use. We were interested in focusing on mental health apps because mental health app use might vary from patterns of physical health app use more generally.

In this paper, we aim to learn more about physical health and mental health app use from the perspective of users with symptoms of depression and anxiety who are interested in exploring such resources for their own benefit. We are specifically interested in learning which kinds of physical and mental health apps these individuals use and what purposes these apps serve for them. By focusing on the apps by use and why, rather than examining the general availability of apps available on the app stores, we may gain greater insight into the health apps people use, the functions of those apps, and the purposes they serve in people’s lives. Ultimately, this information might help shape the design and development of future mHealth tools.

## Methods

### Participants

Android smartphone users were recruited from March 2015 through March 2016 as potential study participants for a field trial of the IntelliCare suite of Android smartphone apps for the treatment of depression and anxiety [16]. Our sample includes people who enrolled in that IntelliCare study, people who were found to be ineligible for that study, and people who were found eligible but ultimately did not enroll. It is worth noting that the common unifying characteristic of the participants is that they were personally seeking Android smartphone apps for treatment of depression or anxiety.

### Recruitment

Participants came from a variety of recruitment sources, with the most common being referrals from a health care organization, Web-based advertising, news stories, advertisements on local public transportation, and word of mouth. The remaining participants came from the Google Play Store, research registries, other research studies, social media, fliers, and other sources.

### Procedures

All potential study participants underwent a multistage screening procedure. First, people completed a brief, 15-minute phone screening with a member of the research staff. If participants were deemed eligible through the phone screening, they were sent the study consent form. When a participant signed the consent form, a member of the research staff reviewed the consent over the phone with the participant, at which point the individual was scheduled for the final eligibility assessment which consisted of completing online questionnaires and a phone interview.

The online questionnaires evaluated symptoms of depression and anxiety using the Patient Health Questionnaire-9 (PHQ-9) [17] and the Generalized Anxiety Disorder-7 (GAD-7) [18], respectively, mental health treatment history, mobile phone use, health app use, and top 5 most used health apps. As we were interested in the apps people had on their Android smartphones before commencing treatment in that study, in this paper we report data from those who partially or fully completed the online questionnaires. Participants were compensated US $20 for completing the online questionnaires. The Northwestern University Institutional Review Board approved all study procedures.

### Eligibility Criteria

Participants were eligible to pass from phone screening to consent form to final assessment if they met the following inclusion criteria:

- PHQ-8 score of 10 or greater [19] or a GAD-7 score of 8 or greater [18] at the time of phone screening
- Owned an Internet-ready Android smartphone with a data package and text plan and were familiar with how to use it
- Were able to speak and read in English
- Were aged 18 years or older (age 19 years in Nebraska, by state law)
- Were US citizens

Participants were excluded at the time of phone screening if they self-reported any of the following:

- Having visual, hearing, voice, or motor impairment that would prevent completion of study procedures
- Diagnosis of psychotic disorder, bipolar disorder, dissociative disorder, or another disorder for which participation was inappropriate or dangerous
- Severe suicidality, including ideation, plan, and intent
- Taking an antidepressant or anxiolytic medication for less than 14 days, not taking a stable dose, or plans to change dose
- Having used any of the IntelliCare apps for a week or more over the past 3 months prior to phone screening

### Measures

#### Online Questionnaires

Participants completed online questionnaires including questions regarding (1) depressive symptoms as indicated by the PHQ-9 [17], a measure that has been shown useful for identifying depression in clinical settings [20,21]; (2) anxiety symptoms as indicated by the GAD-7 [18], a measure that has been shown useful for identifying anxiety in clinical settings [22]; (3) current and past mental health treatment; (4) mobile phone use; (5) health app use; and (6) top 5 most used health apps (see Multimedia Appendix 1 for exact questions developed by the Center for Behavioral Intervention Technologies that were used to query mental health treatment, mobile phone use, health app use, and top 5 most used health apps). Questions were presented to each potential study participant in the same order. The online questionnaires took approximately 30 minutes to complete.

#### Current and Past Mental Health Treatment

This 16-item self-report questionnaire, developed by the Center for Behavioral Intervention Technologies, queried about current and past mental health treatment related to depression and anxiety. Participants responded yes or no to questions such as “Are you currently receiving help for depression?” and “Have you ever sought help for depression?”

#### Mobile Phone Use

This 3-item self-report questionnaire, developed by the Center for Behavioral Intervention Technologies, assessed smartphone use. Participants used a 5-point Likert-type scale, ranging from less than 30 minutes to more than 3 hours, to indicate how much time they spent on their mobile phones on an average day doing the following: calls only, reading (eg, email, text messages, websites, digital books), and using mobile apps.

#### Health App Use

This 14-item self-report questionnaire asked participants to indicate what kind of physical and mental health apps they currently have on their mobile device. They indicated yes or no to 12 categories of physical and mental health apps and had the opportunity to report other types of physical or mental health apps while also reporting the number of health apps they currently had on their smartphones. These categories were constructed through a review, conducted by our research team consisting of clinical psychologists and research staff with experience in eHealth/mHealth, of the types of health apps that are available on app stores and are discussed in the literature. Final categories were reviewed by the team and agreed upon through consensus.

#### Top Five Most Used Health Apps

This 25-item self-report questionnaire, developed by the Center for Behavioral Intervention Technologies, asked participants to enter information on up to 5 physical and mental health apps on their smartphone, selecting the most frequently used apps if they used more than 5. For each app named, participants reported the name of the app, what they used the app for (the app’s purpose), frequency of use (on a 4-point Likert-type scale: multiple times per day, at least once a day, several times per week, or less than once per week), and whether the app was used in the past week (yes/no) or past 24 hours (yes/no).

### Data Review and Coding

#### App Standardization

As an initial step in evaluating the data from the Top 5 Most Used Health Apps questionnaire, one reviewer (CKR) standardized the titles of each health app, referencing the Google Play Store to confirm the correct title, spelling, capitalization, version, and price. For example, Fitbit, fit bit, and FitBit were all coded as the same app (Fitbit) whereas Womanlog and Womanlog Pro were considered 2 distinct health apps due to a difference in app version and price.

All participant-reported apps were assumed to be Android apps accessible via the Google Play Store. In the rare instance when a health app could not be located in the Google Play Store, the reviewer used an Internet search to confirm the title and existence of the health app and its price. In some instances when the reviewer was uncertain whether or not the app was the correct one being referenced, she read through the marketing description of the app in the app store for confirmation.

#### General App Categorization

The mHealth app categories outlined in a report from the IMS Institute for Healthcare Informatics [1] were adapted to characterize the health apps indicated by the participants. The 2 main mHealth app categories were disease and treatment management and wellness management, and the subcategories were health care management, disease-specific, and other for the former main category and fitness, lifestyle and stress, diet and nutrition, and women’s health and pregnancy for the latter main category. Categorizations were completed by 2 reviewers (CKR and SMS), who discussed categories until consensus was reached. Upon discussion, a third category group, nonhealth, was added with the following subcategories: entertainment, productivity, and social to capture apps that were not designed for health purposes but participants reported using for such. All health apps were also coded as either physical heath, mental health, or other.

#### App Purposes

For the participant self-reported app purposes, the first reviewer (CKR) used a thematic grouping qualitative approach, identifying common themes in the free responses to create a set of broad categories [23]. Participant self-reported purposes were closely read, then used to inform the creation of potential categories; once a set of categories was created, the reviewer again went through the original self-reported purposes to ensure categories portrayed the raw text as best as possible. The reviewer went through this kind of iterative process multiple times until all raw text fit neatly into a sensible category and all extraneous categories were removed.

App purpose was determined on a case-by-case basis for each individual participant’s response such that an app could serve different purposes across various participants. By using this end user–centric approach, categories stemmed from the actual use of the app by each participant rather than imposing a predetermined structure to the app purposes based on app store descriptions or content analysis.

#### Developer-Intended App Purposes

Using the finalized set of categories created through qualitative coding of participant self-reports, the first reviewer (CKR) coded each of the participant-mentioned apps based on the brief, marketing material (eg, text and screenshots) found when searching and clicking on that respective app in the Google Play Store. In the rare instance when a health app could not be located in the Google Play Store, the reviewer used an Internet search to locate other marketing material to code the developer-intended purpose.

#### IntelliCare Apps

Since participants were being recruited into a trial to evaluate the IntelliCare app suite for Android devices developed by the Center for Behavioral Intervention Technologies, many of the participants had IntelliCare apps on their Android smartphone at the time of screening as a result of recruitment methods. Thus, we also identified and marked each app that belonged to the IntelliCare app suite because the penetration of IntelliCare apps is likely higher in this population than would be expected in a separate mental health app-seeking population.

### Statistical Analyses

Demographic, clinical, and app characteristics were reported as frequencies and percentages for categorical variables and means and standard deviations for continuous variables. Measures assessing levels of depression and anxiety were reported as means and standard deviations. App characteristics were compared between participants meeting a diagnosis cut-off of depression or anxiety and no diagnosis using a chi-square test for categorical variables. Participants partially or fully completing the online questionnaire pack were included in analyses. Analyses were performed using SPSS versions 23.0 and 24.0 (IBM Corp).

## Results

### Participants

Of the 177 participants who received the online questionnaires, 176 participants partially or fully completed them; 1 participant did not start the questionnaires at all and thus was excluded. Ultimately, a total of 176 participants (135 female, 41 male, mean age 38.64 years, age range 19-75 years) were recruited. All participants were Android users as necessitated by the eligibility criteria to be able to use the IntelliCare suite of apps [16]. Detailed demographic and sample information further characterizing these participants is displayed in Table 1.

**Table 1:** Participant demographic and sample information (N=176).

Variable		Number n (%)
**Age**		
	19-24 years	29 (16.5)
	25-34 years	58 (33.0)
	35-44 years	32 (18.2)
	45-54 years	28 (15.9)
	55-64 years	21 (11.9)
	65+ years	7 (4.0)
	Not reported	1 (0.6)
**Race**		
	African American	16 (9.1)
	Asian	7 (4.0)
	American Indian	1 (0.6)
	Biracial	5 (2.8)
	Native Hawaiian/Pacific Islander	0 (0)
	Not reported/did not identify	3 (1.7)
	White	144 (81.8)
**Ethnicity**		
	Hispanic or Latino	9 (5.1)
	Not Hispanic or Latino	166 (94.3)
	Not reported	1 (0.6)
**Highest level of education**		
	Some high school	1 (0.6)
	Completed high school/general equivancy diploma	6 (3.4)
	Some college	34 (19.3)
	2-year college (associate degree)	18 (10.2)
	4-year college (Bachelor of Arts, Bachelor of Science)	61 (34.7)
	Master’s degree	25 (14.2)
	Doctoral degree	5 (2.8)
	Professional degree (Doctor of Medicine, Juris Doctor)	5 (2.8)
	Not reported	21 (11.9)
**Current employment status**		
	Employed	111 (63.1)
	Unemployed	19 (10.8)
	Disability	11 (6.3)
	Retired	8 (4.5)
	Other	6 (3.4)
	Not reported	21 (11.9)
**Marital status**		
	Single	52 (29.5)
	Married/domestic partner	55 (31.3)
	Separated	2 (1.1)
	Divorced	18 (10.2)
	Widowed	3 (1.7)
	Living with significant other	25 (14.2)
	Not reported	21 (11.9)
**Household income**		
	<$25,000	28 (15.9)
	$25,000-49,999	30 (17.0)
	$50,000-74,999	27 (15.3)
	$75,000-99,999	25 (14.2)
	$100,000-124,999	14 (8.0)
	$125,000-149,999	9 (5.1)
	$150,000+	17 (9.7)
	Not reported	26 (14.8)
**Region of the United States**		
	Northeast (CT, MD, NJ, PA)	8 (4.5)
	Southeast (AL, FL, GA, LA, MS, NC, TN, VA)	14 (8.0)
	Midwest (IA, IL, IN, KS, MI, MN, MO, OH, WI)	128 (72.7)
	Southwest (AZ, NM, TX)	11 (6.3)
	West (CA, CO, HI, OR, UT, WA)	14 (8.0)
	Outside of the United States	1 (0.6)
**Recruitment source**		
	Health care organization	52 (29.5)
	Web-based advertising (eg, Craigslist, Reddit, ResearchMatch)	38 (21.6)
	News stories	23 (13.1)
	Local public transportation advertisements	10 (5.7)
	Word of mouth	10 (5.7)
	Social media (eg, Facebook, Instagram)	5 (2.8)
	Google Play Store	5 (2.8)
	Referral from another research study	3 (1.7)
	Fliers	2 (1.1)
	Illinois Women’s Health Registry	2 (1.1)
	Other sources	26 (14.8)

**Table 2:** Daily mobile phone use (N=176).

Activity		<30 minutes n (%)	30 minutes to 1 hour n (%)	1 to 2 hours n (%)	2 to 3 hours n (%)	>3 hours n (%)
Average time spent for calls only		100 (56.8)	45 (25.6)	10 (5.7)	10 (5.7)	11 (6.3)
Average time spent for reading (eg, email, text messages, websites, digital books)		15 (8.5)	34 (19.3)	43 (24.4)	42 (23.9)	42 (23.9)
Average time spent using mobile apps		24 (13.6)	39 (22.2)	43 (24.4)	38 (21.6)	32 (18.2)

### Depression, Anxiety, and Mental Health Treatment

Participants tended to have moderate levels of depression (PHQ-9 mean score 12.38, SD 5.12, range 2-24) as well as moderate levels of anxiety (GAD-7 mean score 10.76, SD 4.92, range 0-21). The majority of participants (149/176, 84.7%) met the cut-off for a diagnosis of depression or anxiety with 69.9% (123/176) meeting for depression and 69.3% (122/176) meeting for anxiety. Many had sought mental health treatment in the past including 81.8% for depression (144/176) and 67.0% for anxiety (118/176). Many were currently in treatment with 50.6% (89/176) for depression and 40.9% (72/176) for anxiety.

### Mobile Phone and Health App Usage

In total, 77.8% (137/176) of the participants indicated having at least 1 health app on their smartphone, and 86.4% (152/176) of the participants indicated using mobile apps longer than half an hour each day. Table 2 includes information on daily mobile phone use.

On average, participants had 2 health apps on their smartphones (mean 2.15, SD 3.195) with the number of health apps ranging from 0 to 20. The top 3 kinds of physical and mental health apps on people’s phones were exercise, fitness, pedometers or heart rate monitoring apps (93/176, 52.8%), diet, food, or calorie counting apps (65/176, 36.9%), and mental health/wellness apps (46/176, 26.1%). Table 3 includes the types of health apps on participant devices.

### General App Categorization

Only a subset of the participants (107/176, 60.8%) provided more detailed information regarding the specific apps they used and their purposes. Overall, 285 app entries were identified across those 107 users, and 139 unique apps were named.

With regard to the categorizations completed by the 2 reviewers (CKR and SMS), agreement was present for 89 of 139 apps (64.0%). For the remaining 50 apps, the 2 reviewers discussed categories until consensus was reached. Based on the coding of app categories, a majority of the apps (80/139, 57.6%) were for physical health conditions and just over a quarter (39/139, 28.1%) were for mental health. The remaining 20 apps (14.4%) were neither physical health nor mental health apps but apps designed for other purposes that people responded as being health apps that they used. Thus, a third category group, nonhealth, was added with subcategories entertainment, productivity, and social to capture apps that were not technically health apps but that participants indicated using for their health and well-being. Table 4 displays the percentages of apps falling into each category and subcategory divided into physical health, mental health, other, and total.

**Table 3:** Types of health apps currently on mobile phone (N=176). Note: Types of health apps are drawn verbatim from the questionnaire presented to participants (available in Multimedia Appendix 1)

	Yes n (%)	No n (%)	Not reported n (%)
Exercise, fitness, pedometer, or heart rate monitoring	93 (52.8)	82 (46.6)	1 (0.6)
Diet, food, or calorie counter	65 (36.9)	111 (63.1)	0 (0)
Weight	39 (22.2)	137 (77.8)	0 (0)
Period or menstrual cycle	32 (18.2)	144 (81.8)	0 (0)
Blood pressure	6 (3.4)	170 (96.6)	0 (0)
WebMD	23 (13.1)	153 (86.9)	0 (0)
Pregnancy	4 (2.3)	172 (97.7)	0 (0)
Diabetes	0 (0)	176 (100)	0 (0)
Medication management (tracking, alerts, etc.)	17 (9.7)	159 (90.3)	0 (0)
Mood	29 (16.5)	147 (83.5)	0 (0)
Sleep	36 (20.5)	140 (79.5)	0 (0)
Mental health/wellness	46 (26.1)	129 (73.3)	1 (0.6)
Other	21 (11.9)	153 (86.9)	2 (1.1)

**Table 4:** Categories of apps reported.

		Physical health N=80 n (%)	Mental health N=39 n (%)	Other N=20 n (%)	Total N=139 n (%)
**Disease and treatment management**		15 (18.8)	15 (38.5)	0 (0)	30 (21.6)
	Disease-specific	3 (3.8)	14 (35.9)	0 (0)	17 (12.2)
	Health care management	7 (8.8)	0 (0)	0 (0)	7 (5.0)
	Other	5 (6.3)	1 (2.6)	0 (0)	6 (4.3)
**Wellness management**		65 (81.3)	24 (61.5)	0 (0)	89 (64.0)
	Diet and nutrition	13 (16.3)	0 (0)	0 (0)	13 (9.4)
	Fitness	37 (46.3)	0 (0)	0 (0)	37 (26.6)
	Lifestyle and stress	5 (6.3)	24 (61.5)	0 (0)	29 (20.9)
	Women’s health and pregnancy	10 (12.5)	0 (0)	0 (0)	10 (7.2)
**Nonhealth**		0 (0)	0 (0)	20 (100)	20 (14.4)
	Entertainment	0 (0)	0 (0)	3 (15.0)	3 (2.2)
	Productivity	0 (0)	0 (0)	12 (60.0)	12 (8.6)
	Social	0 (0)	0 (0)	5 (25.0)	5 (3.6)

**Table 5:** Top 10 most frequently named apps (N=107).

App name	Code	Number n (%)
S Health	Wellness management: fitness	21 (19.6)
MyFitnessPal	Wellness management: diet and nutrition	21 (19.6)
Fitbit	Wellness management: fitness	15 (14.0)
Thought Challenger^a^	Disease and treatment management: disease specific	12 (11.2)
Daily Feats^a^	Disease and treatment management: disease specific	7 (6.5)
IntelliCare Hub^a^	Disease and treatment management: other	7 (6.5)
WebMD	Disease and treatment management: disease specific	7 (6.5)
Day to Day^a^	Disease and treatment management: disease specific	6 (5.6)
Google Fit	Wellness management: fitness	5 (4.7)
Headspace	Wellness management: lifestyle and stress	5 (4.7)

^a^Apps were part of the IntelliCare app suite.

Table 5 displays the names of the top 10 apps, all of which were at least mentioned by 5 participants.

Of the remaining 129 apps, 2 apps had 4 mentions, 13 apps had 3 mentions, 17 apps had 2 mentions, and 97 apps had 1 mention. Most of the participant-identified apps were free (129/139, 92.8%), with the most expensive app costing $5 and the average cost of downloaded apps being US $0.20 (SD $0.84). Furthermore, 4 of the top 10 apps were part of the IntelliCare app suite (see Table 4).

There were no statistically significant differences between participants who met the threshold for depression or anxiety and those who did not with regard to the types of physical health and mental health apps that they reported downloading. See Multimedia Appendix 2 for additional tables displaying the breakdown of general app categorization and app purposes by diagnosis cut-off for depression or anxiety among the 107 participants who each provided more detailed app information for up to 5 health apps.

### App Purposes

App purpose was tied to each person’s individual use of an app, and Table 6 displays the coding of the app purposes based on the full number of 285 app reports. The most common reason people reported for using these health apps were for different purposes of tracking (117/285, 41.1%).

**Table 6:** Purposes for health app use (N=285).

App purpose		Category description	Number n (%)
**Tracking total**		Track one or more variables	117 (41.1)
	Tracking	Track a single variable	81 (28.4)
	Tracking multiple	Track multiple variables	36 (12.6)
Training/habit building		Long-term training or building a habit	69 (24.2)
Provides routine/activity		Provide exercise or routine	17 (6.0)
Instrument/tool		Used as an instrument or tool	16 (5.6)
Portal		Centralize medical experience, or coordinating a suite of apps	11 (3.9)
Resource		Helpful educational information or information database	11 (3.9)
Multipurpose		Purpose touched on two or more of the other categories	8 (2.8)
No purpose identified yet		App that had been downloaded, but not used enough to report a specific purpose	8 (2.8)
Work		Used for one’s profession	7 (2.5)
Transactional		Functioning as a scheduler or helping fulfill a transaction	6 (2.1)
Entertainment		A game or used to pass time	5 (1.8)
Reminder		Remind user of something	5 (1.8)
Not in Use		Not actually used, or app simply came preloaded on device	3 (1.1)
Community		Socialize or communicate with others	2 (0.7)

Across the 139 distinct apps noted by participants, the majority of those apps (112/139, 80.6%) only had 1 app purpose across the participants who mentioned them, (ie, Headspace was only used for training/habit building across the 5 participants that mentioned it). A notable proportion (27/139, 19.4%) of the apps had 2 or more app purposes across the participants who identified using them (ie, 3 participants indicated using Google Fit for tracking, a fourth participant used it for tracking multiple, and a fifth participant used it as an instrument or tool). Of these 27 apps, 21 apps had 2 app purposes identified, 3 apps had 3 app purposes, and 1 app had 6 app purposes. S Health, the app with 6 app purposes, had the following purposes identified: (1) tracking (pedometer), (2) tracking multiple (tracking steps, heart rate, weight), (3) instrument or tool (heart rate monitor), (4) resource (health information), (5) not in use (I don’t, it’s loaded on my phone), and (6) no purpose identified yet (it came pre-installed on my Samsung Galaxy Note 4).

In terms of usage of these apps, most of the reported apps (188/285, 66.0%) had been used in the past week, and many apps (118/285, 41.4%) had been used within the past 24 hours. This is consistent with reports that participants used these apps frequently with 13.7% of apps (39/285) being used multiple times per day, 22.5% of apps (64/285) being used at least once a day, 24.2% of apps (69/285) being used several times per week, and 39.6% of apps (113/285) being used less than once a week.

One-third of app reports (95/285, 33.3%) were identified as mental health apps, with ultimately 39 distinct mental health apps reported by over half of participants (56/107, 52.3%). The majority of mental health apps reported were used for purposes of training or habit building (57/95, 60.0%) followed by tracking (11/95, 11.6%); the remainder served the following purposes: instrument or tool (8/95, 8.4%), portal (6/95, 6.3%), no purpose identified yet (6/95, 6.3%), provides routine or activity (5/95, 5.3%), tracking multiple (1/95, 1.1%) and not in use (1/95, 1.1%).

Most of the reported mental health apps (67/95, 70.5%) had been used in the past week, and many mental health apps (42/95, 44.2%) had been used within the past 24 hours. This is consistent with reports that participants used mental health apps frequently with 10.5% of mental health apps (10/95) being used multiple times per day, 29.5% of mental health apps (28/95) used at least once per day, 21.1% of mental health apps (20/95) being used several times per week, and 38.9% of mental health apps (37/95) used less than once per week. Just under a quarter of participants (26/107, 24.3%) indicated using mental health apps on a daily basis.

### Developer-Intended App Purposes

The majority of the 139 apps that our participants noted were intended by developers to be multipurpose (80/139, 57.6%). The remaining 59 apps had the following intended purposes: (1) instrument or tool (13/139, 9.35%), (2) training or habit building (12/139, 8.63%), (3) tracking multiple (9/139, 6.47%), (4) resource (6/139, 4.32%), (5) provides activity or routine (5/139, 3.60%), (6) tracking (5/139, 3.60%), (7) community (4/139, 2.88%), (8) portal (2/139, 1.44%), (9) entertainment (1/139, 0.72%), (10) reminder (1/139, 0.72%), and (11) transactional (1/139, 0.72%).

## Discussion

### Principal Findings

This study evaluated health app downloads and use from an end-user perspective by gathering information from participants seeking support for depression and anxiety from an Android mobile app treatment. The results of this study indicated that among this sample, the use of health apps was already quite high. Furthermore, although the specific apps that people downloaded were quite diverse, the purposes for using these apps tended to be quite similar with tracking being the most popular purpose for using a health app. This is further supported by the number of participants using apps where tracking would be a common function such as exercise, fitness, pedometer, or heart rate monitoring; diet, food or calorie counting; menstrual cycle; medication management; mood; and sleep.

Among mental health apps, training or habit building was the most popular purpose that participants indicated for using these specific kinds of apps. Furthermore, while many app developers intended their respective app to be multipurpose, our participants largely used an app for a single purpose. These findings raise questions about health app use and might offer guidelines as to the type of apps and functionality that might be worth developing in the future. Alternatively, these findings might be due to the types of apps currently available on the marketplace, and it might be useful to develop more apps with novel features. This alternative deserves additional consideration in both the design and evaluation of future mobile apps.

Although over three-fourths of participants indicated that they had at least 1 health app on their mobile device, only 60% of our participants actually self-reported the specific apps that they used. It could be that reporting which apps they are using is more difficult than simply endorsing that they are using health apps. In such a case, the answers to this question might better reflect actual use of health apps. Given these were individuals seeking enrollment in a study for the IntelliCare app suite [16], we expected the number of participants reporting health apps to be high. However, our finding that a majority of people are using health apps and that this does not seem to differ between those likely to have clinical levels of distress is similar to findings from other studies [15,24]. There were no statistically significant differences in the kinds of health apps that our participants noted using based on whether they met for depression and/or anxiety or neither. This is consistent with another report [24] finding that healthy individuals and those with chronic conditions may differ minimally when it comes to their use of health apps and the kinds of apps they would be interested in using. Thus, our sample might be generalizable to other health app users.

People reported using health apps often. Almost two-thirds of our sample used a health app at least once per day. Again, this was quite similar to previous findings where nearly two-thirds of participants in 1 study reported opening a health app at least once per day [15]. This is interesting, however, given the overwhelming literature shows low rates of long-term engagement with such tools when examined in the research literature [25,26]. It is possible that (1) consumer apps that are available in the app stores are more engaging despite not being consistently based on empirical research, (2) that people tend to cycle between different apps such that at a given time point they report high use of apps, but use of a single app over time would look much lower, or (3) our sample was unique in that participants were specifically seeking out the IntelliCare apps for their mental health and well-being. Given our methodology, we are unable to disentangle these possibilities, and future studies might consider longitudinal examinations of people’s use of health apps downloaded from commercial stores to learn more about which apps people persist with and which get abandoned. Additionally, the use of health apps continues to grow, and it is possible that health apps have become more accepted and integral parts of people’s lives, which would account for the increased levels of use as compared to data from previously published research literature.

It is worth noting that almost half of the top 10 apps belonged to the IntelliCare app suite, which we believe is a byproduct of this specific group of participants seeking enrollment in a trial of the IntelliCare app suite for Android smartphones [16]. The other apps in the list of top 10 were apps that have been identified as popular health apps in other sources—S Health, MyFitnessPal, Fitbit, WebMD, Google Fit, and Headspace (eg, MyFitnessPal, Fitbit, and WebMD [15]). S Health, one of the apps most frequently identified by our participants, actually comes preloaded on most Android devices and may be a function of that rather than users seeking out health apps. While some participants identified a purpose for S Health, a small number noted that it was merely on their phone but that they did not use it or did not have a purpose for it yet. Ultimately, this again points to the dominance of a relatively few health care apps [1].

Across other reviews of app purposes, the most frequently listed app purposes were some variation of education, treatment or relief, and screening or assessment; tracking (also referred to as symptom monitoring or management) was in the minority of app purposes listed and accounted for less than 10% of health apps [6,7,10]. In our study, however, the substantial majority of app purposes were for tracking. This variation may be a result of the difference in methodological approach, as the reviews had categorized app purposes by looking at apps in consumer app stores, whereas our approach used participant self-reported responses.

On the other hand, one review looking at mental health apps for bipolar disorders in consumer app stores did find that apps with tracking purposes represent a substantial proportion (35/82, 42.7%) of health apps [12]. This same review also showed that the number of resource apps (32/82, 39.0%) was comparable to tracking apps. Interestingly, in our study, while tracking apps were found in similar numbers (53/139, 38.1%) to that review, the number of apps indicated as resources apps by our participants was lower (6/139, 4.3%). Thus, the popularity of tracking apps and not other kinds of apps (eg, resource apps) in our study challenges the notion that participants use tracking apps merely because they are most available in consumer app stores. Instead, end users may be specifically seeking out apps with tracking purposes. By collecting end user self-reported responses, we are better capturing how end users use health apps as opposed to relying solely on information from commercial app stores to inform our understanding of mHealth use.

In terms of the types of apps people download, 3 overwhelming trends emerged: (1) most apps were free, (2) a substantial proportion were used for the purposes of tracking, and (3) mental health apps were most commonly used for purposes of training or habit building. Given that many successful apps on the marketplace tend to focus on doing one thing really well, a future direction for health apps could be to provide a compelling and engaging user experience that builds on tracking and training or habit building for mental health apps specifically. Advances in concepts like self-experimentation and building technologies to help users learn more about links between triggers, behaviors, and symptoms could be a useful starting point to develop this user experience [27]. It is also unlikely that a paid tracking or training or habit-building app would be able to do well in the consumer marketplace given the number of available apps that do this for free, even if not designed specifically for mental health.

A few participants also reported using nonhealth apps for health purposes. This form of app usage is mostly missing when using a strategy that focuses on identifying health apps on the marketplaces or research trials. Other research, however, has suggested several ways in which standard smartphone features or nonhealth apps can serve health purposes. These include alarms [28], Web searches [29], calendar apps [30], and social media [30-32]. This is consistent with some of the apps reported by our participants, such as Google Sheets, which one participant used for migraine tracking. Indeed, nonhealth apps and standard features might have better, more generalizable functionality and better meet user needs. It is also possible health apps that meet user needs and have desired functions do currently exist but users may have a difficult time finding them [15]. Nevertheless, this finding suggests that examining how people use nonhealth apps to support their health care behaviors and needs could prove to be a productive route for researchers to gain new insights for future mHealth tool development.

Our observation that people use tools to manage physical and mental health that were not explicitly designed for those purposes also suggests that incorporating those tools into a digital mental health care system may prove useful. Indeed, general purpose apps may even include functionality consistent with mental health treatment principles. For instance, in April 2016, Google Calendar added a new Goals feature that incorporates principles of behavioral activation. Behavioral activation relies on scheduling and monitoring day-to-day activities and increasing positive activities while bringing awareness to one’s mood and interactions with the environment [33]. The Google Goals feature asks users to choose a personal goal, break that goal into discrete subgoals, and schedule times to complete those subgoals [34]. Goals even helps find open time in the user’s Google Calendar to accomplish these subgoals. Furthermore, Goals uses machine learning to help suggest and schedule goal-related events on a user’s behalf based on that user’s previous pattern of event scheduling. Though Google Calendar with its Goals feature is not necessarily marketed as a health app, the app’s functionality may prove useful for users’ physical and mental health-related goals. Furthermore, as companies expand their range of products and services to meet the needs of their consumers, the distinction is becoming more blurred between health and nonhealth apps.

The authors experienced first-hand the challenges of working with an ever-evolving app marketplace. At times, the authors resorted to Internet searches to find information on participant-mentioned apps that were missing from the Google Play Store. This approach helped the reviewers locate apps that had been renamed or retired from the time participants indicated them to the time the reviewers looked over the data. For example, one participant noted using an app called Skin Deep, which was later integrated into the EWG’s Healthy Living app. At other times, information found in the Google Play Store was misleading. In one instance, an app that was advertised as free, Micromedex Drug Reference, was discovered to be a paid app as noted in the fine print of the description and confirmed by user reviews in the app store. Furthermore, searches for specific app names might bring up numerous other apps because of app store ranking algorithms before the desired apps were to appear. Not surprisingly, other researchers have noted some of these same difficulties [3,11,35]. Thus, researchers who work with app stores should prepare for these kinds of challenges and use alternate methods, such as Internet searches, to assist in finding missing details or supplemental information.

### Limitations

It is worth acknowledging again that the participants in this study were potential participants seeking enrollment in a trial of mental health apps. Thus, these participants are likely not reflective of the general public in terms of their overall mobile health app use and their motivation to use mobile-based mental health resources. Also, most of the participants had sought mental health services in the past and many were still engaged in treatment. This might reflect additional motivation to use services, and health app downloads and usage might be greater than would be reflected in a wider cross-section of the population. Nevertheless, these users may be representative of those who download and use mHealth tools; the apps used and reasons for using them could be particularly informative for the development of new tools. Although participant self-reported data did not support the notion that there were differences in the kinds of physical health and mental health apps on individuals’ phones depending on whether their symptoms met the threshold for depression or anxiety, it is still possible that differences do exist.

Our recruitment strategy focused on Android users (as necessitated by the trial to which this study was linked [16]), thus our participants and their mobile health app use may differ from users of Apple, Windows, or other devices [24]. Certain apps, such as S Health, come preloaded onto Android devices and might be used simply for convenience purposes and would not be present among non-Android users.

In terms of demographic backgrounds, our sample is not representative of the US population. Our sample was largely white and female, with higher levels of income and education, living in the Midwest region of the United States. Nevertheless, certain aspects of demographic information in our sample do seem representative of smartphone users (eg, income, education level) according to one more recent report [36]. Future research should aim to gather a sample representative of the population on multiple demographic levels, including gender, race, ethnicity, income, education, and region.

Finally, we relied on self-report data rather than usage statistics or other passively collected and more objective data such as app downloads. For researchers with a vested interest in the end user experience, participant self-report data may provide information that indicates what kinds of apps people use and the functions those apps serve.

### Future Research

The findings from this study contribute to the evergrowing field of mHealth by exploring health app use reported by users interested in receiving mental health resources via apps. The area of mHealth is still evolving, especially for mental health, and information from all stakeholder groups, including end users, will help build a strong foundation of knowledge. It would be helpful to use more diverse methods (eg, passive data collection such as usage statistics and longitudinal studies) to further explore this space, but this study is a practical first step in learning more about what people download and why. By understanding the kinds of apps individuals are already using, we may be better able to suggest and design other apps that users could also find useful [37].

### Conclusion

This study helps bridge gaps in current knowledge of health app download and use. Reviews of scientific literature and app stores provide important perspectives; however, end user perspectives are also necessary for a more complete and nuanced picture. Furthermore, beginning with end users and what they are able to get on their devices helps mend the discrepancy that exists between the research literature and commercial app marketplaces [11,38]. This allows investigation into the true diversity of health app use that exists, both in terms of the number and types of apps as well as illustrating that people use a variety of different health apps for various reasons to improve their physical health and mental health. Although some purposes appeared to be more popular (eg, tracking or training and habit building) there were numerous purposes that users reported. Expanding the use of end user feedback in the growing research literature will help ensure an app marketplace that can be rigorous, relevant, and responsive.

## Appendix

Multimedia Appendix 1 Screenshots of questions used to query current and past mental health treatment, mobile phone use, and health app use.

Multimedia Appendix 2 Tables displaying breakdown of app purposes and general coding of apps by cut-off for depression and/or anxiety.

## Abbreviations

GAD-7: Generalized Anxiety Disorder Scale-7
PHQ-8: Patient Health Questionnaire-8
PHQ-9: Patient Health Questionnaire-9
