# Supplementary material for: Health App Use Among Individuals With Symptoms of Depression and Anxiety: A Survey Study With Thematic Coding
Source: JMIR Ment Health. 2017 Jun 23;4(2):e22. doi: 10.2196/mental.7603 (PMC5501925; doi:10.2196/mental.7603)
Supplement: Multimedia Appendix 1 [file mental_v4i2e22_app1.pdf]

This is a multimedia appendix to a full manuscript published in JMIR Ment Health, for full copyright and citation information see: http://dx.doi.org/10.2196/mental.7603

Multimedia Appendix

Instructions: Please answer the following questions relating to current and past treatment for depression and/or anxiety.

Are you currently receiving help for depression?	◯ Yes<br>◯ No
Are you currently receiving help for anxiety?	◯ Yes<br>◯ No
Are you currently receiving help from a Primary Care Phsyician?	◯ Yes<br>◯ No
Are you currently receiving help from a Psychiatrist?	◯ Yes<br>◯ No
Are you currently receiving help from a Psychologist?	◯ Yes<br>◯ No
Are you currently receiving help from a another Mental Health Professional?	◯ Yes<br>◯ No
Are you currently receiving help from another provider not listed above? If so, please specify below.	◯ Yes<br>◯ No
Other (specify):	______________________
Are you currently taking any prescription medication for anxiety or depression (e.g., Prozac, Effexor, Zoloft, Paxil)?	◯ Yes<br>◯ No
Have you ever sought help for depression?	◯ Yes<br>◯ No
Have you ever sought help for anxiety?	◯ Yes<br>◯ No
Did you seek help from a Primary Care Physician?	◯ Yes<br>◯ No
Did you seek help from a Psychiatrist?	◯ Yes<br>◯ No
Did you seek help from a Psychologist?	◯ Yes<br>◯ No
Did you seek help from a another Mental Health Professional?	◯ Yes<br>◯ No
Did you seek help from another provider not listed above? If so, please specify below.	◯ Yes<br>◯ No
Other (specify):	______________________
Have you ever taken any prescription medication for anxiety or depression (e.g., Prozac, Effexor, Zoloft, Paxil)?	◯ Yes<br>◯ No

Instructions: Please answer the following questions on the use of your smartphone.

	Less than 30 minutes	From 30 minutes to 1 hour	From 1 to 2 hours	From 2 to 3 hours	More than 3 hours
1. How much time do you spend on your mobile phone on average in a day (calls only)?	○	○	○	○	○
2. How much time do you spend on your mobile phone on average in a day reading (e.g. email, text messages, websites, digital books, etc.)?	○	○	○	○	○
3. How much time do you spend on your mobile phone on average in a day using mobile apps?	○	○	○	○	○

4. Please indicate what kind of health and mental health apps do you currently have on your phone.

	Yes	No
Exercise, fitness, pedometer or heart rate monitoring (includes specific types of exercise like running, ab workouts, yoga, etc.)	○	○
Diet, food, calorie counter	○	○
Weight	○	○
Period or Menstrual Cycle	○	○
Blood Pressure	○	○
WebMD	○	○
Pregnancy	○	○
Blood Sugar or Diabetes	○	○
Medication management (tracking, alerts, etc.)	○	○
Mood	○	○
Sleep	○	○
Mental Health/Wellness	○	○

Do you use any other kinds of health or mental health apps not listed above? If so, please describe below.

○ Yes
○ No

Please describe: ______________________________

5. How many health apps do you currently have on your phone?

____
(If you have not used any apps for your mood and mental health, please enter 0.)

Enter information for up to 5 health and mental health apps below. Please select the apps you've used the most. If you've used more than 5 apps, enter info for the 5 you use most frequently:

App #1:

App Name: ________________________________

What do you use this app for? (What's the purpose of the app?): ________________________________

Frequency:
- ◯ Multiple times per day
- ◯ At least once a day
- ◯ Several times per week
- ◯ Less than once per week

	Yes	No
Did you use this app in the past week?	◯	◯
Did you use this app in the past 24 hours?	◯	◯

App #2:

App Name: ________________________________

What do you use this app for? (What's the purpose of the app?): ________________________________

Frequency:
- ◯ Multiple times per day
- ◯ At least once a day
- ◯ Several times per week
- ◯ Less than once per week

	Yes	No
Did you use this app in the past week?	◯	◯
Did you use this app in the past 24 hours?	◯	◯

App #3:

App Name: ______________________________

What do you use this app for? (What's the purpose of the app?): ______________________________

Frequency:
- ◯ Multiple times per day
- ◯ At least once a day
- ◯ Several times per week
- ◯ Less than once per week

	Yes	No
Did you use this app in the past week?	◯	◯
Did you use this app in the past 24 hours?	◯	◯

App #4:

App Name: ______________________________

What do you use this app for? (What's the purpose of the app?): ______________________________

Frequency:
- ◯ Multiple times per day
- ◯ At least once a day
- ◯ Several times per week
- ◯ Less than once per week

	Yes	No
Did you use this app in the past week?	◯	◯
Did you use this app in the past 24 hours?	◯	◯

App #5:

App Name: ______________________________

What do you use this app for? (What's the purpose of the app?): ______________________________

Frequency:
- ◯ Multiple times per day
- ◯ At least once a day
- ◯ Several times per week
- ◯ Less than once per week

	Yes	No
Did you use this app in the past week?	◯	◯
Did you use this app in the past 24 hours?	◯	◯
