# Supplementary material for: Health App Use Among Individuals With Symptoms of Depression and Anxiety: A Survey Study With Thematic Coding
Source: JMIR Ment Health. 2017 Jun 23;4(2):e22. doi: 10.2196/mental.7603 (PMC5501925; doi:10.2196/mental.7603)
Supplement: Multimedia Appendix 2 [file mental_v4i2e22_app2.pdf]

This is a multimedia appendix to a full manuscript published in JMIR Ment Health, for full copyright and citation information see: http://dx.doi.org/10.2196/mental.7603

Multimedia Appendix

Table 7. App purpose endorsement by diagnosis cut-off

App Purpose	Participants, n(%) (N = 107)	Participants Who Met Depression, n(%) (N = 77)	Participants Who Met Anxiety, n(%) (N = 75)	Participants Who Met Depression and/or Anxiety, n(%) (N = 92)
Tracking Total	73 (68.2%)	50 (64.9%)	48 (64.0%)	62 (67.4%)
Tracking	55 (51.4%)	35 (45.5%)	36 (48.0%)	46 (50.0%)
Tracking Multiple	30 (28.0%)	25 (32.5%)	22 (29.3%)	27 (29.3%)
Training/Habit Building	47 (43.9%)	33 (42.9%)	33 (44.0%)	41 (44.6%)
Provides Routine/Activity	15 (14.0%)	10 (13.0%)	9 (12.0%)	12 (13.0%)
Instrument/Tool	15 (14.0%)	11 (14.3%)	10 (13.3%)	12 (13.0%)
Portal	10 (9.3%)	7 (9.1%)	7 (9.3%)	8 (8.7%)
Resource	11 (10.3%)	6 (7.8%)	9 (12.0%)	9 (9.8%)
Multipurpose	7 (6.5%)	7 (9.1%)	5 (6.7%)	7 (7.6%)
No purpose identified yet	5 (4.7%)	4 (5.2%)	5 (6.7%)	5 (5.4%)
Work	2 (1.9%)	0 (0.0%)	0 (0.0%)	0 (0.0%)
Transactional	6 (5.6%)	3 (3.9%)	3 (4.0%)	3 (3.3%)
Entertainment	2 (1.9%)	2 (2.6%)	2 (2.7%)	2 (2.2%)
Reminder	5 (4.7%)	3 (3.9%)	3 (4.0%)	4 (4.3%)
Not in Use	3 (2.8%)	3 (3.9%)	3 (4.0%)	3 (3.3%)
Community	1 (0.9%)	1 (1.3%)	1 (1.3%)	1 (1.1%)

Note: These data reflect the proportion of participants who reported using one or more apps in the stated categories. Participants who had PHQ-9 scores greater than or equal to 10 met for depression, and participants who had GAD-7 scores greater than or equal to 8 met for anxiety. The denominator in the second column (N = 107), represents the number of participants who chose to provide more detailed information for up to five specific health apps they used and their purposes. The denominator in the third column (N = 77), represents the number of the 107 participants who met for depression; the fourth column denominator (N = 75), represents the number of the 107 participants who met for anxiety; the fifth column denominator (N = 92), represents the number of the 107 participants who met for depression and/or anxiety.

Table 8. Categories of apps endorsement by diagnosis cut-off

Category of Apps	Participants, n(%) (N = 107)	Participants Who Met Depression, n(%) (N = 77)	Participants Who Met Anxiety, n(%) (N = 75)	Participants Who Met Depression and/or Anxiety, n(%) (N = 92)
DTM: Disease Specific	37 (34.6%)	23 (29.9%)	26 (34.7%)	32 (34.8%)
DTM: Healthcare Management	10 (9.3%)	7 (9.1%)	7 (9.3%)	8 (8.7%)
DTM: Other	9 (8.4%)	6 (7.8%)	7 (9.3%)	8 (8.7%)
WM: Diet & Nutrition	31 (29.0%)	19 (24.7%)	23 (30.7%)	25 (27.2%)
WM: Fitness	59 (55.1%)	43 (55.8%)	39 (52.0%)	50 (54.3%)
WM: Lifestyle & Stress	39 (36.4%)	27 (35.1%)	23 (30.7%)	31 (33.7%)
WM: Women's Health & Pregnancy	13 (12.1%)	8 (10.4%)	9 (12.0%)	11 (12.0%)
NH: Entertainment	3 (2.8%)	2 (2.6%)	3 (4.0%)	3 (3.3%)
NH: Productivity	8 (7.5%)	6 (7.8%)	4 (5.3%)	6 (6.5%)
NH: Social	1 (0.9%)	1 (1.3%)	1 (1.3%)	1 (1.1%)

Note: These data reflect the proportion of participants who reported using one or more apps in the stated categories. Participants who had PHQ-9 scores greater than or equal to 10 met for depression, and participants who had GAD-7 scores greater than or equal to 8 met for anxiety. The denominator in the second column (N = 107), represents the number of participants who chose to provide more detailed information for up to five specific health apps they used and their purposes. The denominator in the third column (N = 77), represents the number of the 107 participants who met for depression; the fourth column denominator (N = 75), represents the number of the 107 participants who met for anxiety; the fifth column denominator (N = 92), represents the number of the 107 participants who met for depression and/or anxiety. DTM = Disease and Treatment Management. WM = Wellness Management. NH = Non-Health.
